# A case of orthodontic splint ingestion misclassified as a sharp/pointed foreign body in a child: A case report

**DOI:** 10.1097/MD.0000000000041843

**Published:** 2025-04-25

**Authors:** Hyun Kyung Lee, Jung Hee Byon, Eun Hae Park

**Affiliations:** a Department of Radiology, Jeonbuk National University Medical School and Hospital, Jeonju, Republic of Korea; b Research Institute of Clinical Medicine of Jeonbuk National University-Biomedical Research Institute of Jeonbuk National University Hospital, Jeonju, Korea; c Department of Radiology, Ulsan University Hospital, University of Ulsan College of Medicine, Ulsan, Republic of Korea.

**Keywords:** foreign body, ingested, orthodontic, wire

## Abstract

**Rationale:**

Foreign body (FB) ingestion is common, particularly among children. While most FBs pass through the gastrointestinal tract without complications, some cases can lead to morbidity or even mortality. Management strategies depend on the size, sharpness, toxicity, and location of the FB. Plain radiographs are essential for diagnosis, treatment planning, and follow-up, especially for radiopaque FBs. Ingestion of dental-related FBs is rare, with reported cases involving orthodontic wires, braces, retainers, and other dental materials. Composite resin-wire splints are commonly used in orthodontics to manage dental traumatic injuries. We present a case of a patient who ingested a composite resin-wire splint, initially misclassified as a sharp FB, leading to unnecessary aggressive treatment.

**Patient concerns:**

A 2-year-old girl presented to the emergency department after accidentally ingesting a FB of dental origin. She had a recent traumatic dental injury and displayed no symptoms of ingestion. Initial radiographs revealed a dental wire in the stomach.

**Diagnoses:**

Ingestion of orthodontic splint (composite resin-wire splint).

**Interventions:**

Considering the pointed tip of the wire, the emergency department physician transferred her to the endoscopy specialist for endoscopic removal. However, given the expected passage into the small bowel after a proper fasting period for endoscopy, close observation with follow-up radiographs was chosen amid parental anxiety.

**Outcomes:**

Follow-up imaging showed FB migration to the ascending colon without perforation. Stool inspection revealed the dental wire and 4 composite resins, with the sharp tips covered by the resin, classifying it as a blunt FB. A retrospective review of radiographs revealed faint oval-shaped, mid-level radiopacities at each end of the wire covering the sharp/pointed tips.

**Lessons:**

In evaluating orthodontic splints as FBs, identifying faint composite resin on radiographs is crucial for planning milder treatment and alleviating parental anxiety.

## 1. Introduction

Foreign body (FB) ingestion is relatively common in childhood, with the majority of incidents occurring between 6 months and 3 years of age. Most FBs (80%–90%) in the gastrointestinal (GI) tract pass spontaneously without complications, while 10% to 20% require endoscopic removal, and 1% necessitate open surgery due to complications.^[1]^ The ingestion of sharp/pointed FBs, such as pins, nails, screws, needles, or orthodontic prostheses, is associated with high morbidity and mortality. Delayed diagnosis and treatment can significantly increase the risk of severe complications, including perforation, extraluminal migration, abscess formation, or peritonitis. To prevent potential complications from sharp/pointed FBs, endoscopic retrieval is recommended whenever they are accessible via endoscope.^[2,3]^ The incidence of FB ingestion of dental origin in the general population varies considerably in the literature, ranging from 3.6% to 27.7% of all FB ingestions.^[4,5]^ Cases involving the ingestion of orthodontic wires or fragments from braces, retainers, and other dental materials – whether occurring in or outside the orthodontic office – have been documented, but they remain relatively rare compared to more commonly ingested objects.^[6]^ Plain radiographs play a crucial role in assessing FB ingestion, particularly for radiopaque objects. Radiographs assist in identifying the type of FB, its location within the GI tract, and the presence of associated complications. In cases of ingested FB of dental origin, it is well established that radiography is essential for locating, tracking, and retrieving them.^[7,8]^ Here, we present a case of accidental ingestion of an orthodontic splint, where the mid-level radiopacity of the composite resins was overlooked and misclassified as a needle-like sharp FB, resulting in an aggressive treatment plan, accompanied by a review of the relevant literature.

## 2. Case report

A 2-year-old girl was brought to the emergency department (ED) for FB ingestion. She had a history of dental subluxation injury to the left mandibular lateral incisor tooth following trauma, which was treated with orthodontic splinting 2 days prior. She swallowed the orthodontic splint accidentally while drinking milk. At the time of evaluation, she exhibited no clinical signs of airway obstruction or clinical symptoms such as drooling, chest discomfort, or chest pain.

**Figure 1.**
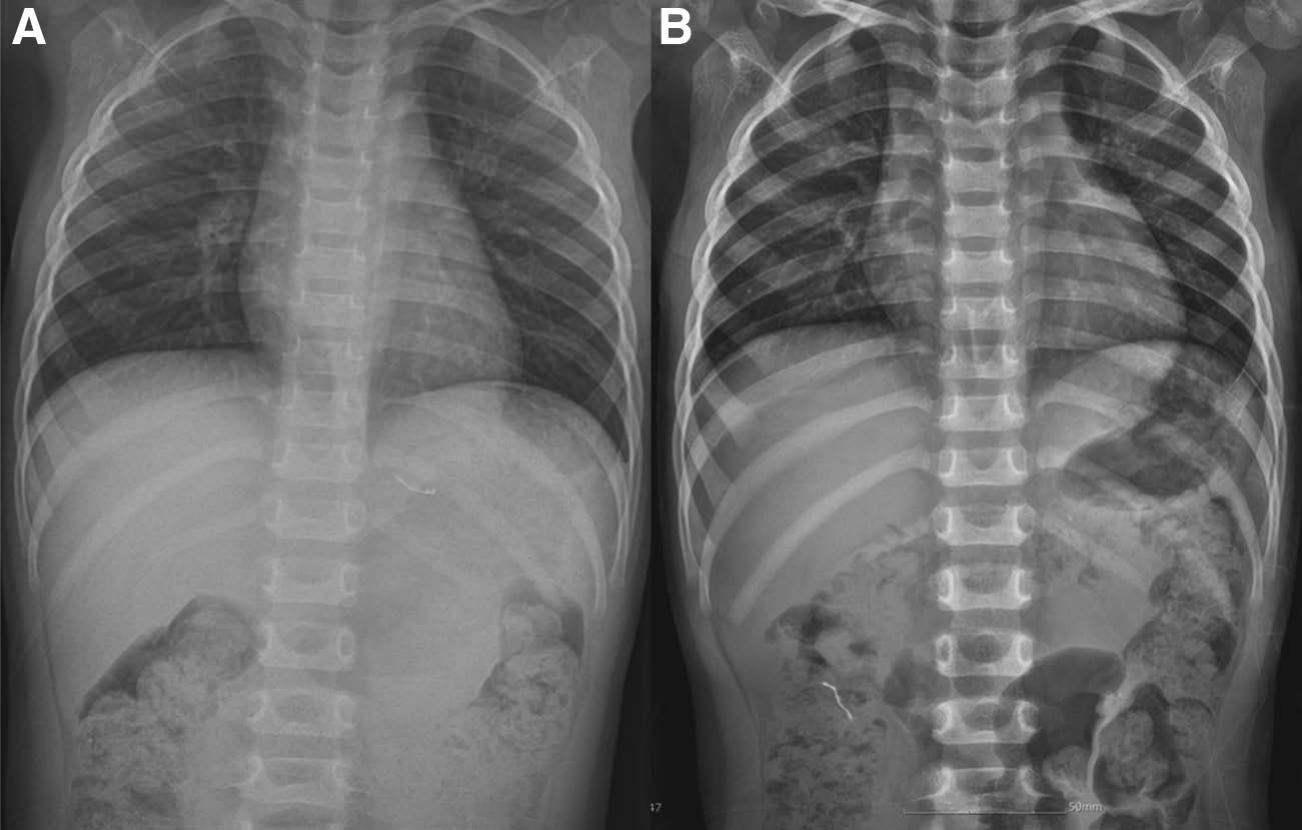
(A) Chest radiographs in the ED revealed a swallowed orthodontic splint in the stomach. (B) A follow-up radiograph obtained 11 h later showed the orthodontic splint in the ascending colon without any signs of intestinal perforation or associated complications. ED = emergency department.

**Figure 2.**
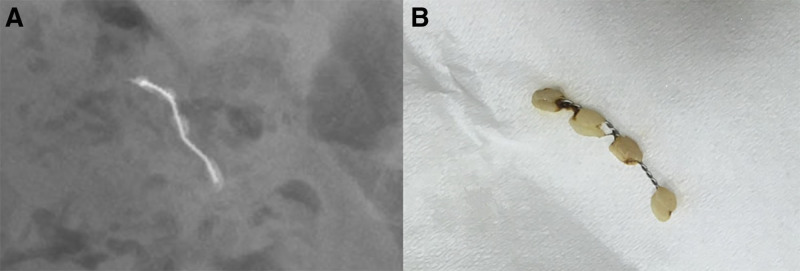
(A) Stool inspection identified the composite resin-wire splint, with the blunt ends covered by resin composites. (B) A magnified radiograph revealed an orthodontic wire measuring 1.5 cm, accompanied by 4 composite resin pieces, which appeared as oval-shaped radiopaque objects.

The initial chest radiograph obtained in the ED showed a 1.5 cm-sized orthodontic wire with sharp tips in the stomach (Fig. 1A). The patient was transferred to an endoscopic specialist for FB removal due to the risk of intestinal perforation associated with the sharp/pointed tip of the orthodontic wire. However, the physician recommended observation instead of endoscopic removal, as the orthodontic wire was anticipated to pass beyond the duodenal curve after the fasting period required for endoscopy. Observation was decided with the consent of her parents, despite their considerable anxiety. A follow-up radiograph obtained 11 hours after ingestion revealed the orthodontic wire in the ascending colon (Fig. 1B). One day after this follow-up radiograph, approximately 24 hours post-ingestion, a wire with composite resins was discovered during stool inspection (Fig. 2A). The orthodontic wire splint successfully passed through the GI tract in 24 hours.

The orthodontic splint discovered during stool inspection had 4 intact composite resin pieces, that were covering both sharp/pointed ends of the wire, which indicated that it was a blunt object rather than a sharp/pointed one. Upon a retrospective review of the 2 radiographs with magnification (Fig. 2B), the resins appeared as oval-shaped radiopacities at each end of the wire, effectively covering the sharp/pointed ends. The patient was discharged later that day without any adverse events.

## 3. Discussion

Foreign body ingestion is more prevalent in the pediatric population than in adults. In children, it typically occurs accidentally with common household objects, whereas in adults, it may result from suicide attempts, psychiatric disorders, intellectual disabilities, or secondary gain. The management of FBs remains controversial but is generally guided by factors such as size, sharpness, chemical properties, and the location within the GI tract.^[1,2]^ In the present case, the FB was initially identified as a sharp wire with a needle-like edge. The clinical significance of sharp/pointed FB ingestion arises from its higher complication rate compared to other types of FBs, which can lead to increased morbidity and mortality. Previous studies have indicated that while the overall rate of intestinal perforation due to FB ingestion is <1%, this rate escalates to 15% to 35% for sharp/pointed FBs.^[9,10]^

Although there is currently no consensus or established treatment guidelines, endoscopic retrieval is generally recommended within the first 24 hours for asymptomatic sharp/pointed FBs found in the stomach, provided the patient is nil per os (NPO).^[2]^ Early diagnosis and prompt endoscopic removal have been demonstrated to reduce the incidence of related complications.^[3,11]^ For this reason, endoscopy was considered the first-line treatment in the present case. However, retrieval was ultimately declined, as the FB was expected to pass into the small bowel after ensuring proper NPO conditions. Instead, clinical follow-up with serial radiographs was conducted, with physicians exercising caution and advising parents about potential complications, which understandably caused them anxiety.

The follow-up proved to be appropriate, as it was determined that each end of the wire was covered with resin, resulting in blunt margins rather than needle-like sharp edges. Asymptomatic, nontoxic blunt FBs found in the stomach are expected to pass spontaneously; however, elective endoscopy is recommended if they do not pass within 2 to 4 weeks.^[2]^ A study reports the mean transit time for ingested FBs in children to be 3.6 day.^[12]^ In this case, the ingested FB was expelled in just 2 days, slightly earlier than the average.

Plain radiographs play an important role in assessing FB ingestion in pediatric patients, not only for initial diagnosis but also for follow-up. They can confirm the presence of most FBs, particularly radiopaque ones, and help identify the type of object, its location within the GI tract, and any associated complications. Additionally, radiographs can reveal risk factors for complications, such as sharp or wide FBs. If a radiographically detected object is deemed likely to pass without intervention, serial imaging is performed to monitor its timely progression and elimination.^[7,13,14]^ However, the opacity of FBs may vary. Due to unfamiliarity with the details of the technique and the less-opaque nature of resin, the resin covering the wire was initially overlooked, leading to the misconception that the FB had needle-like margins. In retrospect, 4 composite resins were faintly but clearly depicted on the radiographs.

The incidence of FB ingestion from dental origins in the general population varies considerably in the literature, ranging from 3.6 to 27.7 percent of all FBs, which is more common in adults than children.^[4,5]^ There is a risk of swallowing removable prostheses or orthodontic parts during and after treatment. Composite resin-wire splints, which typically involve fixation with a flexible wire and composite resin, are among the most commonly used splints for managing traumatic dental injuries.^[15]^ However, due to the hydrophilic nature of resins, they are prone to water sorption and hydrolytic degradation in the oral environment.^[16]^ This creates a risk of bond failure at the resin-dentin interface, potentially leading to the ingestion or inhalation of orthodontic wires, as seen in the present case.

Restorative dental materials, including resin, should have optimal radiopacity to clearly differentiate the restoration-tooth interface from the surrounding tooth structure. A radiopacity level slightly higher than that of enamel, often referred to as mid-level opacity, is preferred for evaluation on radiographs.^[17,18]^ The International Standards Organization (ISO) defines radiopacity standards, stating that restorative dental materials applied to coronal tooth tissue should have radiopacity equal to or greater than that of the same thickness of pure aluminum.^[19]^ This means that composite resins meeting ISO standards are radiopaque and therefore visible on X-rays.

Image contrast on radiographs is primarily influenced by tube voltage (Kilovoltage peak, kVp); A low kVp produces a short grayscale with high contrast image, whereas a higher kVp produces a long grayscale with lower contrast image.^[20]^ The visibility of FBs on radiographs, especially those with intermediate and low radiopacity, can vary depending on the kVp settings used. This visibility is enhanced by utilizing a low kVp technique, with settings in the range of 65 to 70 kVp.^[7]^ In our case, the tube voltage was set at 85 kVp, allowing the composite resin to be relatively clearly visible. Detecting the resin component in the FB in the present case may have altered the treatment plan and reduced parental anxiety. Further studies are needed to investigate the detection sensitivity of various types of restorative dental materials on non-dental X-rays.

## 4. Conclusion

This is a case report of an accidental ingestion of an orthodontic splint that occurred outside the orthodontic office, describing its passage through the GI tract without any complication. Recognizing these objects and their faint visibility on radiographs can aid in correctly classifying the FB as a blunt object rather than a sharp/pointed one, enhancing decision-making confidence and alleviating anxiety for both patients and their guardians in cases of various FB ingestion from dental origin.

## Author contributions

**Conceptualization:** Jung Hee Byon, Eun Hae Park, Hyun Kyung Lee.

**Data curation:** Jung Hee Byon, Eun Hae Park, Hyun Kyung Lee.

**Investigation:** Jung Hee Byon.

**Supervision:** Eun Hae Park, Hyun Kyung Lee.

**Validation:** Eun Hae Park.

**Writing – original draft:** Jung Hee Byon, Eun Hae Park, Hyun Kyung Lee.

**Writing – review & editing:** Jung Hee Byon, Eun Hae Park, Hyun Kyung Lee.
